# Mercury in Scrofulous Affections of the Lungs

**Published:** 1844-04

**Authors:** 


*Mercury in Scrofulous Affections of the Lungs.*—Dr. Graves makes some valuable, because practical, remarks on the effects of mercury in scrofulous affections of the lung. He was first led to the adoption of this mode of treatment by the successful results of mercury in the hands of Dr. O'Beirne in treating scrofulous inflammation of the joints. This employment of mercury in the treatment of scrofulous diseases was directly in the teeth of the prevailing theories of the day, according to which mercury was not only inadmissible, but absolutely mischievous in persons of a scrofulous diathesis. Dr. Graves was led by analogy to apply the same principle of treatment to incipient scrofulous inflammation of the lung. He points out the cases to which such treatment is applicable; it is, he says, only in those cases wherein scrofulous inflammation commences in the lung before any general contamination of the system has taken place, that mercury ought to be tried, and it will be of no avail except where the commencement of the scrofulous inflammation of the lung has arisen suddenly, and in consequence of the operation of some obvious cause, as catching cold, or the occurrence of hæmoptysis. Dr. Graves thinks that too much stress has been laid on the affection of the lung by writers on phthisis. In most cases, no doubt, the disease commences in the lung; but, in other cases, it passes through many changes, and affects various organs before it attacks the lung. Dr. Graves has a decided objection to the terms, "tubercular inflammation," and considers the whole theory of inflammation being excited in the lung by the presence of tubercles, to be founded on erroneous views. He considers, also, that tubercles do not act in all cases on foreign bodies. He thinks that, in the treatment of scrofulous bronchitis and scrofulous pneumonia, mercury is a most valuable remedy—where the attack is recent, and has occurred under circumstances which preclude any suspicion of previous tubercular disease.—*Med. Chi. Rev.*, Jan.

